# Milk Yield and Quality, Metabolic Profile and Oxidative Status in Lactating Goats, as Affected by Silage Based on Agro-Industrial By-Products

**DOI:** 10.3390/ani16030500

**Published:** 2026-02-05

**Authors:** Angela Gabriella D’Alessandro, Abdelfattah Z. M. Salem, Giovanni Martemucci

**Affiliations:** Department of Soil, Plant and Food Sciences, University of Bari, 70126 Bari, Italy; abdelfattah.salem@uniba.it (A.Z.M.S.);

**Keywords:** agro-industrial by-products, dairy goats, by-product silage, milk quality, oxidative status, metabolic profile, polyphenols

## Abstract

Large amounts of agro-industrial residues—such as grape pomace, olive mill wastewater, wheat straw, and cheese whey—pose disposal challenges and generate substantial environmental and economic burdens. Incorporating these by-products into ruminant diets may reduce feeding costs while supporting sustainability within a circular bioeconomy. Two experiments were conducted to evaluate: (i) the characteristics of a mixed raw agro-industrial by-product silage (BPS) in cylindrical bale silos (50 kg) and (ii) its effects as dietary supplementation on metabolic profile, oxidative status, milk yield, and milk quality in lactating goats. The BPS was formulated from raw wheat straw, grape pomace, olive mill wastewater, and cheese whey and tested at a 30% inclusion level (dry-matter basis). The combined by-products produced in 50 kg cylindrical bale silos were positively characterized and exhibited a nutritionally suitable silage with relevant antioxidant potential. Dietary BPS improved oxidative status, as evidenced by increased TAS and vitamin E and reduced ROMs, and positively influenced lipid and immunological blood markers of goats. Milk yield was higher in goats receiving the BPS. Moreover, milk quality was improved through a more favorable fatty acid profile, higher vitamin E content, reduced cholesterol levels, and enhanced oxidative stability, as evidenced by lower TBARS values. Overall, BPS supplementation enhanced animal health indicators and milk nutritional properties, demonstrating that this silage can contribute to more sustainable dairy goat production while offering potential benefits for human nutrition.

## Simple Summary

The use of agro-industrial wastes such as grape pomace, olive mill wastewater, wheat straw, and cheese whey in animal feed represents a valuable strategy to reduce environmental impact and promote sustainable livestock production within a circular bioeconomy framework. In this study, a mixed silage produced in cylindrical bale silos (50 kg) from these combined raw agro-industrial residues was included in the diet of lactating dairy goats. The supplementation improved milk yield and enhanced several quality traits, including: a more favorable fatty acid profile, characterized by higher levels of polyunsaturated fatty acids and conjugated linoleic acid (CLA), milk oxidative stability, metabolic status and animal health. Overall, the results demonstrate that incorporating agro-industrial by-product silage into goat diets can improve animal performance, enhance the nutritional value of milk, and support sustainability by reusing materials that would otherwise contribute to environmental waste.

## 1. Introduction

In recent decades, increasing political and social pressure to reduce environmental impact has driven changes in agro-industrial production processes, enabling the reuse of their residues [1] within the context of a circular bioeconomy focused on sustainable biomass utilization, based on waste valorization and the optimization of biomass value over time [2]. The circular bioeconomy is guided by the biomass value pyramid, in which animal feed ranks second in terms of biomass value, prioritizing the most valuable uses over energy generation and composting [3]. Within this framework, the use of agro-industrial by-products in ruminant feeding allows their conversion into high-quality foods for human consumption, such as milk and meat [4,5,6], while simultaneously promoting the development of a circular economy, reducing land and water use for food production, limiting competition between human and animal nutrition, and lowering the overall environmental impact [7,8].

In the Mediterranean area, straw, olive mill wastewater (OMWW), grape pomace (GP), and cheese whey are among the main agro-industrial by-products, derived from various supply chains: cereals (straw), olive oil (OMWW), wine (GP), and dairy (whey). Although straw provides limited carbohydrates, nutritional value, and bioactive compounds, the other by-products are generally rich in carbohydrates, proteins, minerals, lipids, and bio-functional compounds [9], particularly polyphenols. Their composition makes them promising ingredients for livestock diets, offering nutritional benefits and potential health-promoting properties that can affect ruminant productivity and health [6,10,11], as well as enhance the physicochemical properties and oxidative stability of milk [3,12,13].

Goat milk consumption ranks third worldwide [14] and continues to increase, particularly in less-favored areas and in the Mediterranean basin [15,16], due to its nutritional composition. It is a significant source of nutrients in the human diet, including polyunsaturated fatty acids (PUFAs) [17,18], which may contribute to the prevention of cancer and cardiovascular and metabolic diseases [19,20], and can serve as an alternative to cow’s milk for individuals with allergies to this food [21].

Straw, a by-product of cereal cultivation, is commonly used as fodder for ruminants and as bedding in animal husbandry. However, in many regions, most straw is burned, causing environmental pollution through CO_2_ emissions [22].

In the Mediterranean area, over 30 million m^3^ of OMWW is produced annually, representing 95–97% of global production [23,24]. OMWW, composed of fruit water and processing water, is characterized by high organic pollution, with a chemical oxygen demand (COD) up to 220 g O_2_ L^−1^ and a COD/biochemical oxygen demand (BOD_5_) of 2.5 and 5. Its discharge represents a significant environmental concern [25], as it is often released directly into soils and water bodies through uncontrolled disposal practices, thereby posing a serious threat to ecosystems [26]. The composition of OMWW includes a low pH (3.5–5.5) and a high polyphenol content of up to 80 g L^−1^, along with notable antioxidant activity [27].

Grape pomace is a by-product of wine production, representing approximately 25% of the grape mass. It includes stalks, skins, pulp, and seeds. Improper disposal of GP in open areas can lead to environmental issues by contamination of soil and groundwater [28]. GP contains numerous polyphenols that exhibit strong antioxidant activity [29,30], due to their incomplete extraction during wine processing [31].

Cheese whey, the liquid by-product of cheese production, accounts for over 80% of milk used in dairy industry [32]. It exhibits a high pollution load, with a BOD ranging from 27 to 60 kg m^−3^, a COD from 50 to 102 kg m^−3^, and a BOD_5_/COD ratio typically exceeding 0.5 [33]. Whey retains approximately 55% of the milk’s nutrients and around 20% of its total protein content [34].

The incorporation of agro-industrial by-products into animal feed is increasingly recognized as a viable strategy to reduce the environmental footprint of livestock production. This practice provides an alternative to costly waste management while lowering feed expenses, particularly for ruminants raised under extensive or semi-intensive systems in hot–arid regions where pasture availability is highly dependent on unpredictable seasonal conditions. Although the intrinsic variability in the nutrient composition of these by-products [4] offers potential advantages for their valorization in silage formulations, by allowing adjustments in ingredient composition to ensure optimal fermentation and nutritional targets, their irregular and seasonal availability constitutes a major limitation for large-scale use [35].

Ensiling has emerged as an effective method to preserve nutritional attributes and stabilize bioactive constituents, including polyphenols, thereby enabling their efficient incorporation into ruminant diets. Several studies have investigated the use of OMWW, GP, wheat straw, and whey in silage formulations intended for beef cattle, lambs, and pigs [36,37,38].

Dietary grape pomace supplementation in ruminants generally improves milk quality by reducing total saturated fatty acids (SFAs) and increasing monounsaturated fatty acids (MUFAs) and polyunsaturated fatty acids (PUFAs), as well as bioactive fatty acids such as vaccenic and rumenic acids [6,39]. Similarly, dietary supplementation with OMWW in dairy sheep enhances the nutraceutical value of milk and cheese [40]. The inclusion of whey and wheat straw in silage fed to beef cows has been shown to positively affect productive performance and feed efficiency [36]. More recently, silages prepared using mixtures of maize grains, OMWW, GP, and cheese whey after undergoing preliminary processing have been explored [41,42,43].

In a previous study [44] conducted at the laboratory scale, we investigated the potential of using wheat straw together with grape pomace, olive mill wastewater, and cheese whey as raw ingredients for by-product-mixed silages. No studies have examined the combined use of these by-products for silage production in field silos to assess whether the changes occurring during the silage process better reflect real conditions, which are less controlled than laboratory-scale experiments. Moreover, there is a lack of research investigating the effects of such multi-by-product silage on milk yield, milk composition, and health status of lactating ruminants.

Dairy goats are exposed to high metabolic demands during lactation, which can compromise productive efficiency [42]. Dietary supplementation with GP, rich in polyphenols, has been shown to improve milk quality in ruminants, including dairy cows [5,10], while feed supplemented with phenolic extracts derived from OMWW has improved the nutritional profile of sheep milk [45]. We hypothesized that dietary supplementation with a silage enriched in polyphenol-containing by-products could enhance animal health by improving oxidative balance and could promote the transfer of polyphenol compounds to milk, improving its quality in dairy goats. Accordingly, the objectives of this study were to: (i) characterize the silage (BPS) produced from agro-industrial by-products—wheat straw, grape pomace, olive mill wastewater, and cheese whey—as raw ingredients prepared in baled form (50 kg cylindrical bale) to evaluate its quality and suitability as ruminant feed (Experiment 1) and (ii) evaluate its effects, when provided as a dietary supplement, on blood biochemical parameters, oxidative status, milk yield, and milk composition in dairy goats within a sustainability-oriented framework for improving dairy production systems (Experiment 2).

## 2. Materials and Methods

### 2.1. Silage Preparation and Analysis

#### 2.1.1. Silage Preparation

The by-products used were wheat straw (*Triticum durum*; WS); grape pomace (GP) from the vinification of *Vitis vinifera* L. (cv. Primitivo); olive mill wastewater (OMWW) from olive milling (*Olea europaea*, cv. Ogliarola); and cheese whey (CW) obtained from bovine milk dairy processing. Agro-industrial farms located in the Apulia region of southern Italy provided the by-products, which were refrigerated at 2–4 °C until use (12–24 h). Experimental silage production was carried out on a private farm in the same region. Based on previous studies, the proportion of raw ingredients was established to obtain a fresh-matter ratio of 60% solids (WS, 40% + GP, 20%) and 40% liquids (CW, 35% + OMWW, 5%) [44]. Thirty cylindrical silage bales each weighing approximately 50 kg were produced. Silage was prepared using a mixing wagon: WS was chopped to an average length of 3–5 cm and mixed with the other by-products (GP, OMWW, and CW). A commercial freeze-dried starter culture of *Lactobacillus plantarum* (14/DCSL CECT 4528; 250 × 10^9^ cfu/g; Lactosil, CSL Zelo Buon Persico, Lodi, Italy) was used as a silage additive. After thorough mixing, the ingredient blend was compacted and wrapped with four layers of bale-wrap plastic using a bale wrapper machine. At ensiling (D0), a sample of the mixture was taken prior to bale packaging for analysis. The silage bales were stored for 40 days (maturation period) and subsequently analyzed. For the analyses, ~1 kg of silage was collected from each of three different bales using a manual auger inserted into three different areas of each bale. Subsamples from each bale were combined to form three replicates. In the laboratory, each replicate was divided into three portions: one for pH, dry matter, and fermentation characteristics; one dehydrated (60 °C, 48 h); and one frozen at −20 °C for chemical composition and total phenolic content analyses.

#### 2.1.2. Analyses of Chemical Composition and Fermentative Characteristics of the Silage (BPS)

The silage was analyzed for chemical composition, fermentation characteristics, total phenolic content and quality.

Ground silage samples (1 mm) were analyzed using standard AOAC [46] methods to determine dry matter (DM; method 950.46), crude protein (CP; method 990.03), ether extract (EE; method 920.39), and ash (method 920.153). Neutral detergent fiber (NDF), acid detergent fiber (ADF), and acid detergent lignin (ADL) were determined according to Van Soest et al. [47].

Total phenolic content of the silage was determined using the Folin–Ciocalteu assay [48]. Briefly, phenolics were extracted from 200 mg lyophilized silage with methanol:water (80:20, *v*/*v*, 1% formic acid) by sonication (10 min) and centrifugation (15,000 rpm, 15 min, 4 °C). The extraction was repeated under the same conditions. Supernatants were combined and filtered. The filtrate was then centrifuged at 2000 g for 10 min, evaporated under vacuum at ≤40 °C, and dissolved in methanol and stored at −30 °C until analysis. For quantification, 100 µL of extract was mixed with the Folin–Ciocalteu reagent (Sigma-Aldrich, St. Louis, MO, USA) and 20% Na_2_CO_3_ and incubated for 60 min at room temperature. Absorbance was measured at 760 nm with a spectrophotometer (Cary 60 UV-Vis Spectrophotometer, Agilent, Santa Clara, CA, USA). Total phenolic concentration was expressed as milligrams of gallic acid equivalents (GAEs) per gram of silage dry matter (DM).

For pH determination, 10 g of silage was mixed with 90 mL of distilled water for 5 min. pH was measured using a WTW InoLab pH Level 3 m (Xylem Analytics Germany Sales GmbH & Co. KG, WTW, Weilheim, Germany). Two replicates were performed. Silage quality was assessed using the Flieg point system, calculated according to [49]:

Flieg’s score = 220 + (2 × %DM − 15) − 40 × pH

Silage quality was classified as: very good (score 81 to ≥100), good (61–80), medium (41–60), inferior (21–40), or very inferior (<20).

Lactic acid concentration was determined spectrophotometrically at 570 nm (Cary 60 UV-Vis Spectrophotometer, Agilent, Santa Clara, CA, USA) according to Madrid et al. [50]. Volatile fatty acid (VFA) concentrations—acetic, propionic, and butyric acids—were determined using a gas chromatograph (Agilent 7890A GC, Agilent Technologies Italia Spa, Rome, Italy) equipped with a capillary column (SACtm-5, 300 cm × 0.25 mm; Supelco, Bellefonte, PA, USA). Quantification was performed using an external calibration method based on a commercial SCFA standard mixture (Volatile Free Acid Mix, Supelco, Bellefonte, PA, USA). Individual SCFAs were identified by comparing retention times with those of authentic standards. The sum of total VFAs (acetic, propionic, and butyric acids), total acids (VFA plus lactic acid), and their relative proportions (total VFA/total acids) were calculated.

### 2.2. In Vivo Trial

#### 2.2.1. Animals, Experimental Design, and Diets

The animals used in the experiment to evaluate how silage by-products affect milk yield and quality, as well as animal welfare, were Ionica lactating goats from a private farm located in the Apulia region (Southern Italy). The goats of each experimental group (*n*. 15) were housed in a single pen having an indoor surface area of 30 m^2^ (2 m^2^/goat), with straw bedding, with access to outdoor yards, and with free access to water and mineral blocks. Animals were fed and milked twice daily by mechanical milking, at 09:00 and 18:00. The experiment was carried out during the spring season.

Thirty Ionica goats in their third month of lactation (body weight 47.4 ± 4.7 kg; average milk yield 1.2 ± 0.7 kg/day) were randomly assigned to two dietary groups corresponding to the experimental treatments: CON (control diet, without silage by-products) and BPS (diet including agro-industrial by-product silage). The control diet included oat hay (*Avena sativa*), soybean meal 44%, corn flour, and a vitamin–mineral premix. The experimental diet included 30% silage by-products on a dry matter basis; the remainder consisted of hay, soybean meal, corn flour, and a vitamin–mineral premix. Table 1 reports the ingredient composition of each diet and their chemical composition. The in vivo trial lasted 50 days. During the first two weeks, the animals were allowed to adapt to their respective diets.

#### 2.2.2. Sample Procedures and Analyses

The chemical composition of the diets was analyzed as previously described for silage, following AOAC [46] procedures for DM, EE, CP, and CF. NDF, ADF, and ADL were analyzed according to Van Soest et al. [47].

During the last three days of the trial, milk yield was recorded, and milk samples were collected from both morning and evening milkings for subsequent laboratory analyses. Fecal and blood samples were collected from all animals on the second day of this three-day period.

Fecal samples were collected directly from the rectum of each animal using sterile gloves and immediately stored for short-chain fatty acid (SCFA) analysis, according to Ribeiro et al. [52], with slight modifications.

Approximately 10 g of feces was homogenized in 50 mL of deionized water (1:5, *w*/*v*) and centrifuged at 3000× *g* for 15 min, and the supernatant was filtered through Whatman No. 1 filter paper (Sigma-Aldrich, Darmstadt, Germany). SCFAs were stabilized with metaphosphoric acid (25%, *w*/*v*), shaken for 10 min, and extracted with toluene. SCFAs were quantified by gas chromatography with flame ionization detection (Agilent 7890A GC, Agilent Technologies Italia Spa, Rome, Italy) using a capillary column (SAC™-5, 300 cm × 0.25 mm; Supelco, USA). SCFAs were identified and quantified based on standard elution times using a commercial SCFA standard mixture (Volatile Free Acid Mix, Supelco, Bellefonte, PA, USA).

Individual milk samples were collected in standard 100 mL bottles, refrigerated at 4 °C, and transported to the laboratory for chemical analyses. Pooled morning and afternoon milk samples were analyzed for fat, total protein, lactose, and casein using an IR spectrophotometer (MilkoScan 133B, Foss Electric, Hillerød, Denmark). All analyses were performed in duplicate. Somatic cell count (SCC) was determined using a Fossomatic 90 cell counter (Foss Electric) according to the IDF [53] method.

Milk fatty acids were analyzed according to Folch et al. [54]. Fatty acids were trans-esterified using sodium methoxide in methanol [55] and quantified using a Trace GC 2000 gas chromatograph equipped with a flame ionization detector (ThermoQuest, CE Instruments, Rodano (MI), Italy). Fatty acids were separated on a silica capillary column (SP-2560, 100 m × 0.25 mm; Supelco Inc., Bellefonte, PA, USA). Helium was used as the carrier gas. Individual fatty acid methyl esters were identified by comparison with an internal standard (methyl-nonadecanoate; Sigma Chemical Co., St. Louis, MO, USA). A commercial CLA isomer standard mixture containing cis-9, trans-11 and trans-10, cis-12 C18:2 (Sigma Chemical Co.) was used for peak identification. The two isomers were separated based on retention time, and quantification was performed exclusively for the cis-9, trans-11 isomer (rumenic acid). Milk cholesterol content was determined according to Fletouris et al. [56] using capillary gas chromatography. Samples (0.2 g) were saponified in capped tubes with 0.5 M methanolic KOH at 80 °C for 15 min. Water was added, and unsaponifiable fractions were extracted with hexane and analyzed by gas chromatography.

Vitamins A and E in milk samples were extracted and analyzed by HPLC according to Zhao et al. [57]. The HPLC system was equipped with an autosampler (HPLC Autosampler 360, Kontron Instruments, Milan, Italy; 20 µL loop), a high-pressure mixing pump, and a C18 column (5 µm, 250 × 4.60 mm; Phenomenex, Torrance, CA, USA). Peak areas were quantified using Kroma System 2000 software (v. 1.8.1) against reference standard curves.

Total phenolic content in milk was determined using the Folin–Ciocalteu colorimetric method. For extraction, 7 mL of acetonitrile with 3% (*v*/*v*) formic acid was added to 1 mL of milk. The mixture was stirred (3 min), sonicated (10 min), and centrifuged (4000 rpm, 10 min, 4 °C). After storage at −20 °C overnight, the supernatant was collected, centrifuged again (14,000 rpm, 10 min, 4 °C), filtered, and stored at −20 °C until TPC determination. Total phenolics were analyzed according to Singleton et al. [48], as previously described for the silage.

Lipid peroxidation in milk was assessed by determining TBARS levels according to Manglano et al. [58]. Absorbance was measured at 532 nm, and results were calculated using a standard curve prepared with 1,1,3,3-tetramethoxypropane (Sigma-Aldrich, St. Louis, MO, USA). TBARS were expressed as micromol thiobarbituric acid per liter of milk.

The effects of the diets on goat metabolism were assessed by collecting blood samples from the jugular vein into vacutainer tubes on the second day of milk. Blood biochemical parameters were determined using an automated biochemical analyzer (TC-220 TECOM, Jiangxi, China) with commercial kits following standard colorimetric procedures.

Serum concentrations of triglycerides (TG; 505 nm), total cholesterol (TC; 505 nm), high-density lipoprotein cholesterol (HDL-C; 570 nm), alkaline phosphatase (ALP; 405 nm), and aspartate aminotransferase (AST) were measured using diagnostic kits (SPINREACT, Sant Esteve de Bas, Girona, Spain). Alanine aminotransferase (ALT; 340 nm) was determined using a commercial kit (PRO-EKO, Petacciato, Campobasso, Italy).

Inflammatory cytokines (TNF-α, IL-1β, and IL-6) were measured using test kits from Immunological Sciences (Rome, Italy) following the manufacturer’s instructions, using an Infinite TECAN M1000Pro microplate reader (Tecan, Männedorf, Switzerland).

The oxidative status of the animals was evaluated by measuring total antioxidant status (TAS), reactive oxygen metabolites (ROMs), and vitamins A and E.

TAS was measured spectrophotometrically according to Erel [59], and the results were expressed as Trolox equivalents (TE/L). ROM values were determined using a colorimetric commercial kit (Diacron, Grosseto, Italy) at 505 nm according to the manufacturer’s instructions [60] and expressed in Carr units (1 U/Carr = 0.024 mmol/L H_2_O_2_).

Vitamin A and E concentrations in blood samples were analyzed using the same HPLC method described for milk samples. Retinol and α-tocopherol were extracted from plasma with chloroform, and detection was performed using a fluorometric detector with two wavelengths (vitamin A: 325 nm; vitamin E: 300 nm). Retinyl acetate and α-tocopherol acetate were used as internal standards, and concentrations were expressed as mg/100 mL of plasma.

### 2.3. Statistical Analysis

Data on the characteristics of the agro-industrial by-product silage, milk yield and quality parameters, metabolic profile, and oxidative status of the animals were analyzed using statistical software (SPSS for Windows, version 23.0, IBM Corp., Armonk, NY, USA). For variables measured over three consecutive days, the mean value per animal was calculated to obtain a single observation for statistical analysis. Differences between the experimental groups were assessed using an independent-samples *t*-test. Differences were considered statistically significant at *p* < 0.05, while *p* values between 0.05 and 0.10 were considered trends.

## 3. Results

### 3.1. Chemical Composition and Fermentative Characteristics of Silage (BPS)

The chemical composition and fermentative characteristics of the agro-industrial by-product silage are reported in Table 2; VFA data correspond to the time at which the ensiling process reached maturity (D40). Dry matter (DM) and the chemical components of the experimental silage at D0 showed a slight reduction (*p* > 0.05) at maturation (D40). At D40, the BPS was characterized by a relatively high DM content (50.42%). Ash, crude fiber, and fiber fractions (NDF, ADF, and ADL) also appeared unaffected by the silage formulation, showing only slight reductions during ensiling (*p* > 0.05). The total polyphenol content (TPC) of the BPS at D0 was 1.62 mg GAE g^−1^ DM, reflecting the TPC of GP and OMWW (14.40 ± 0.21 vs. 5.21 ± 0.36 mg GAE g^−1^ DM, respectively). At maturation (D40), TPC significantly increased (*p* < 0.01) to 2.57 ± 0.021 mg GAE g^−1^ DM. As expected, the pH of the BPS was higher at D0 (5.59) and decreased during ensiling, reaching 3.99 at D40 (*p* < 0.01). Among VFAs, lactic acid showed the highest concentration (26.50 g kg^−1^ DM), followed by acetic, propionic, and butyric acids. The Flieg score, expressing silage quality, was 146.64 at D40.

### 3.2. Milk Yield and Components and Fecal Short Chain Fatty Acids (SCFAs)

The effects of the dietary inclusion of BPS on milk production and composition are shown in Table 3. Milk yield significantly increased in the BPS group compared with the CON group (*p* < 0.05). No diet-related differences were observed for protein, fat, or lactose content, while milk cholesterol concentration was significantly reduced (*p* < 0.01) in the BPS group. Somatic cell count (SCC) was also lower (*p* < 0.05) in the BPS compared to CON.

The total SCFA concentration was lower in the CON group. In the BPS group, acetate represented the largest proportion of total fecal SCFAs. Propionic and butyric acid concentrations were significantly higher in BPS-fed goats than in CON (*p* < 0.001).

### 3.3. Milk Fatty Acid Profile

Table 4 reports the effects of the BPS on SFAs, MUFAs, PUFAs, and CLA in milk fat. Saturated fatty acids (SFAs) represented the largest FA category in milk and were significantly lower (*p* < 0.05) in BPS-fed goats. Dietary treatment did not affect (*p* > 0.05) the concentrations of short- and medium-chain FAs (butyric 4:0, caproic 6:0, caprylic 8:0, and capric 10:0). In BPS goats, palmitic acid decreased (*p* < 0.05) and stearic acid increased (*p* < 0.05) compared to CON. No differences were found for total MUFAs (*p* > 0.05), which were predominantly composed of oleic acid (18:1 cis-9). However, the BPS significantly increased PUFA levels (*p* < 0.01) and resulted in higher concentrations (*p* < 0.05) of C18:2 cis-9,trans-11 (CLA), C18:3 n-3 (α-linoleic acid, ALA), C20:5 n-3 (eicosapentanoic acid, EPA), and C22:6 n-3 (docosaesanoic acid, DHA) while C20:4 n-6 (arachidonic acid) showed a tendency (0.05 < *p* < 0.10) to increase.

### 3.4. Blood Metabolic and Immunologic Profiles

The effects of BPS supplementation on blood biochemical, lipid, and immunological parameters and liver activity in goats are shown in Table 5. No significant dietary effects were observed for total protein, triglycerides, ALP, AST, or ALT (*p* > 0.05). Total cholesterol decreased and HDL concentrations increased in the BPS group (*p* < 0.05).

Dietary BPS exerted an immunomodulatory effect, significantly reducing TNF-α and proinflammatory IL-1β levels (*p* < 0.05). IL-6 concentration showed a non-significant reduction (*p* > 0.05) in BPS goats compared with CON.

### 3.5. Antioxidant Activity in Blood and Milk

The effects of BPS supplementation on the oxidative status of goats are presented in Table 6. BPS-fed goats exhibited higher blood total antioxidant status (TAS) (*p* < 0.05) and lower plasma reactive oxygen metabolites (ROMs) (*p* < 0.05) compared with CON. Vitamin E levels were also higher (*p* < 0.05) in BPS goats, while vitamin A did not differ between groups (*p* > 0.05).

In milk, the BPS resulted in a higher (*p* < 0.05) total phenol and vitamin E content, as well as lower TBARS values (*p* < 0.05), whereas vitamin A levels were not affected.

## 4. Discussion

### 4.1. Chemical Composition and Fermentative Characteristics of Silage (BPS)

The use of agro-industrial wastes in animal nutrition is a key strategy to mitigate environmental impact and promote sustainable livestock systems within a circular bioeconomy. In this study, a silage was formulated from raw by-products of cereal, olive oil, wine, and dairy supply chains and validated as a dietary ingredient for lactating goats. Wheat straw was incorporated for dietary fiber; cheese whey for its caloric, lactose and nutrient content [61]; GP for its phenolic compounds, unsaturated fatty acids, dietary fiber, and beneficial microorganisms [25,26]; and OMWW for the presence of several antioxidant compounds with high radical scavenging activity, including hydroxytyrosol, oleuropein, tyrosol, caffeic acid, p-coumaric acid, verbascoside and elenolic acid [62,63].

Although these agro-industrial by-products have been individually tested in several trials previously, this is, to our knowledge, the first study evaluating a silage that combines four raw agro-industrial by-products (WS, OMWW, GP, and CW) in the diet of lactating goats.

DM is considered an essential factor of nutritional preservation after ensiling, including protein content [64]. During ensiling, DM losses occur through effluents and gas production [65]. To minimize excessive nutrient losses, Massaro et al. [66] reported that the optimal initial DM content in grape pomace silage may range between 280 and 400 g/kg DM. In the present study, the DM content was relatively higher, which may be due to the characteristics of the raw materials, but this did not appear to adversely affect the fermentation process, as indicated by satisfactory pH values and silage quality parameters. In the present study, DM and other chemical components of the experimental silage remained almost unchanged between D0 and D40, indicating efficient nutrient retention. The maintenance of DM content may be attributed to the homo-fermentative activity of lactic acid bacteria (LAB), which convert glucose into lactate without inducing DM loss [67].

Phenolic compounds have strong antioxidant activity that contributes substantially to the antioxidant potential of feed [68]. Polyphenols play a crucial role in improving the overall quality and nutritional value of silage. The stability depends on their chemical structure and the matrix composition that improves or mitigates degradation processes [69]. Contrary to previous reports describing a reduction in polyphenols during ensiling [70,71,72], in this study an increase in TPC was observed. This increase may be associated with the activity of β-glucosidase-producing lactic acid bacteria, which has been attributed to the release of phenols during silage fermentation, making them more accessible to the solvent during extraction and, consequently, increasing the measured antioxidant potential of silage [73].

Silage pH is a key indicator of fermentation quality, and a final pH between 3.8 and 4.2 denotes successful ensiling [69], as observed in this study. The low pH reflects efficient fermentation and the inhibition of undesirable microorganisms such as clostridia, enterobacteria, *Listeria* spp., and molds, whereas a pH above 5 promotes microbial deterioration and reduces silage quality [74,75]. These results can be attributed to the activity of the starter culture of *Lactobacillus plantarum* associated with the total phenol content of OMWW and GP present in BPS and their probable synergistic action, as observed between phytogenic feed additives [76]. In particular, OMWW showed a higher TPC than GP, as previously reported [44].

Fermentation products strongly influence the hygienic and nutritional quality of silage. Lactic acid was the predominant VFA in this study, supporting optimal pH reduction and ensuring a good concentration of nutrients and palatability [69]. The lactic:acetic ratio is considered a good indicator of fermentation and silage quality, with the best levels ranging between 3 and 1 [74]. In this study, the lactic:acetic ratio also fell within the recommended range (3–1), indicating high fermentative efficiency. Acetic acid usually represents the second concentration in silage [69], as in this study; it is considered useful for inhibiting yeast and improving its stability in the air. Acetic acid can be adsorbed from the rumen, is utilized as an important source of metabolizable energy and represents a major precursor for de novo fatty acid synthesis in the mammary gland, thereby contributing to milk fat production [74,77,78].

Propionic acid may be undetectable in silages with DM contents greater than 35%, as observed in this study, whereas it is commonly present in very wet silages (<25% DM) [69]. High levels of propionic acid (>0.3–0.5%) have been associated with poorly fermented silages and/or with the presence of certain *Clostridia* strains [79]. Although propionic acid is a key gluconeogenic precursor absorbed from the rumen and used to support hepatic gluconeogenesis, excessive levels in silage typically reflect broader fermentation issues that may compromise animal performance [80,81,82].

Butyric acid was detected at a level of 1.98 g/kg DM, which is close to the acceptable threshold of 2.0 g/kg DM [83]. High dietary butyric acid has been associated with increased production of ketone bodies and a higher risk of subclinical ketosis in lactating dairy cows [84,85]. The concentration of total volatile fatty acids and the ratio of volatile fatty acids to total acids are indicators of fermentation efficiency. In this study, the concentration of total volatile fatty acids was close to 20%, a value considered adequate [86].

The Flieg score, based on the pH and dry-matter content, is commonly used as an index to classify the quality of silage. In this study, the BPS exhibited excellent silage quality, indicating that the silage formulation had high quality and stability [49,87], as evidenced by the Flieg score being higher than 100. Overall, the results indicate that ensiling the combination of raw agro-industrial by-products—WS (40%), GP (20%), OMWW (5%), and CW (35%)—in cylindrical bale silos is a practical and promising technique, as it preserves their nutritional composition over time. It should also be emphasized that the baled silage format is efficient for handling and transporting silage feed, simplifying logistics. Additionally, it offers storage flexibility, maximizing the use of available farm space [88].

### 4.2. Milk Yield and Components and Fecal Short Chain Fatty Acids (SCFAs)

In this study, the inclusion of 30% (dry matter) BPS in the total ration increased milk yield in the BPS group compared with the control group. Dietary supplementation with substances rich in phenolic compounds—such as silymarin [89], grape pomace [90], or artichoke silage [91]—has been reported to improve milk yield in goats, whereas other studies reported no effect of grape pomace on milk production in goats and sheep [92], nor olive cake silage in sheep rations [93]. Differences in milk response may depend on the level of inclusion, chemical characteristics, and physical form of the by-products. No improvements in milk yield were observed in Churra ewes fed grape pomace [94], whereas increased milk yield was reported in dairy cows supplemented with grape marc [95]. Similarly, Moate et al. [96] observed higher milk yield in cows fed dried rather than ensiled grape pomace. Overall, the effects of phenols on milk in small ruminants remain inconsistent [9], likely due to variations in phenolic concentration and source, potential associative or antagonistic interactions among phenolic compounds [12,76], and differences in polyphenol bioavailability [97,98].

In our study, the inclusion of 30% BPS (DM basis) in goat diets did not affect milk macro-composition, suggesting that the overall nutritional adequacy of the diet was maintained. The decrease in milk cholesterol in the BPS group may be related to the phenolic content of OMWW and GP, in agreement with other studies on phytogenic dietary supplementation rich in polyphenols in goats [99,100].

The lower somatic cell counts observed in the BPS group may also be related to the high polyphenol content of OMWW and GP. Phenolic compounds are known to exert bactericidal effects in the mammary gland by inhibiting major mastitis-related pathogens such as *Staphylococcus aureus*, *Escherichia coli*, and *Klebsiella pneumoniae*, which are directly related to SCC levels [101].

Fecal SCFAs were evaluated to assess the potential effects of BPS on intestinal function. SCFAs are the main products of intestinal microbial fermentation of dietary fiber and carbohydrates through distinct metabolic pathways [102]. In this study, acetate was the predominant SCFA, followed by propionate and butyrate, in agreement with previous findings [103]. These SCFAs support intestinal barrier function [104], participate in carbohydrate and lipid metabolism [105], and serve as energy substrates [106,107]. Acetate is a major precursor for fatty acid synthesis [77], whereas propionate can be used for gluconeogenesis [108].

Acetate and propionate are generally associated with a higher abundance of *Bacteroides* spp., whereas butyrate production is linked to *Firmicutes* [109]. In our study, propionic and butyric acid concentrations were significantly higher in the BPS group than in the CON. This increase may be attributed to the modulatory effects of polyphenols present in grape pomace and OMWW on gut microbiota composition and metabolic activity, which are known to enhance microbial fermentation efficiency and SCFA production [110,111]. The polyphenol content of OMWW has been shown to promote the growth of beneficial bacterial populations, such as *Lactobacillus* spp. and *Bacillus* spp., while inhibiting pathogenic bacteria [63]. Similarly, grape pomace supplementation has been reported to increase beneficial microbial populations and SCFA production in the lower intestine [112,113]. These mechanisms may collectively explain the higher propionate and butyrate levels observed in BPS-fed goats.

### 4.3. Milk Fatty Acid Profile

The milk lipid profile is influenced by diet [9], and agro-industrial by-products such as grape pomace, olive mill wastewater, and cheese whey contain significant amounts of bio-functional components, including polyphenols, dietary fiber, and unsaturated fatty acids. Dietary silage formulated with these agro-industrial ingredients has been shown to improve the fatty acid profile of meat in piglets and broilers [37,114]. In this study, the milk lipid profile was improved by including the BPS in the goats’ diet, probably because of the presence of grape pomace and OMWW, resulting in lower SFA values and increased PUFAs and CLA. The reduction in SFAs disagrees with other research on dietary grape pomace supplementation in goats and cows [10,92]. The concentrations of short- and medium-chain fatty acids — butyric (4:0), caproic (6:0), caprylic (8:0), and capric (10:0)—were not affected by BPS treatment, representing a positive outcome. In particular, butyric acid is associated with health benefits [115], while caproic and capric acids may improve milk fat digestibility and contribute to the sensory quality of milk and dairy products [116,117]. The lack of change in fatty acids synthesized almost exclusively de novo in the mammary gland (C6–C14) suggests that dietary BPS does not negatively affect carbohydrate fermentation in the rumen [118]. The observed lower palmitic acid (16:0) content in milk may indicate a potential reduction in enteric methane emissions in dairy goats, consistent with previous reports [119]. Stearic acid is the final product of ruminal biohydrogenation [120]. The observed increase in stearic acid in BPS goat milk could be attributed to enhanced ruminal biohydrogenation from C18:1 to C18:0 [121]. Several trials suggest that 18:0 is beneficial or at least neutral for cardiovascular disease prevention and does not show negative health effects compared with other SFAs [122].

No differences in MUFA content were found between treatments, consistent with findings following the inclusion of grape pomace in the diet of small ruminants [92,123]. Goats fed the BPS diet exhibited a higher content of cis-9, trans-11 CLA, the main milk CLA isomer, derived from Δ9-desaturase activity and ruminal biohydrogenation [110]. Various human studies have demonstrated beneficial effects of CLA on atherosclerosis and related markers, as well as reduced incidence and severity of diabetes, obesity, immune dysfunction, and cancer [121].

The total PUFA content in milk fat was influenced by dietary BPS, in agreement with results reported in ewes, cows, and goats fed grape pomace [5,92,124] and with studies on OMWW supplementation in ewes and goats [45,125]. BPS supplementation increased the levels of C18:3 n-3 (ALA, alpha-linolenic acid), C20:5 n-3 (EPA, eicosapentaenoic acid), and C22:6 n-3 (DHA, docosahexaenoic acid), while C20:4 n-6 (arachidonic acid) showed a tendency to increase. This effect can be explained by the action of polyphenols in BPS, which reduce or inhibit the activity of rumen microorganisms responsible for PUFA biohydrogenation [126], limiting the conversion of ALA to saturated fatty acids. A higher dietary phenolic content may thus promote ALA accumulation [127], which serves as a precursor for long-chain n-3 fatty acids such as EPA and DHA. Overall, the findings suggest that a diet supplemented with BPS containing OMWW and grape pomace polyphenols helps preserve these PUFAs in milk, resulting in an enrichment of health-promoting fatty acids. Long-chain n-3 PUFAs are essential for human health due to their beneficial effects in reducing the risk or severity of several chronic diseases [128].

### 4.4. Blood Metabolic and Immunologic Profiles

Blood represents a major indicator for evaluating the physiological, nutritional, and general pathological status of animals. Environmental and/or physiological stressors can induce oxidative stress, leading to an accumulation of free radicals in cells and tissues. This condition arises from an imbalance between free radicals and antioxidant molecules in the body, in favor of free radicals, and can be counteracted by antioxidants [129,130].

Dietary supplementation with BPS affected several blood parameters. The decrease in cholesterol levels could be due to the inhibition of dietary cholesterol absorption in the intestine, reduced hepatic cholesterol synthesis, or the stimulation of biliary secretion and fecal excretion of cholesterol [131,132].

The liver enzymes ALP, AST, and ALT showed no significant differences between experimental groups, indicating that dietary BPS exerted neutral effects on hepatic injury. Conversely, the BPS reduced the levels of the proinflammatory cytokines TNF-α and IL-1β, suggesting a beneficial influence on immunomodulatory responses, likely due to the action of polyphenols present in GP and OMWW [63,133].

Oxidative stress is directly linked to inflammation, since ROS activate NF-κB, a key regulator of inflammatory processes. However, polyphenols from OMWW and GP can inhibit the production of various inflammatory molecules both in vitro and in vivo [63,133]. Miliaraki et al. [134], in their analysis of oxidant/antioxidant pathways, reported that the expression of the cytokines TNF-α and IL-6 was strongly correlated with an increase in TAS, in agreement with our findings.

### 4.5. Antioxidant Activity in Blood and Milk

Dietary BPS treatment positively affected the oxidative status markers TAS, ROMs, and vitamin E, likely due to the OMWW and GP components in the BPS, and possibly to their synergistic action. The antioxidant activity of OMWW is mainly attributed to hydroxytyrosol (10–25%), flavonoids (6–19%), and secoiridoid derivatives (13–27%) [27], whereas the antioxidant activity of GP is attributable to phenolic compounds, including gallic acid, p-coumaric acid, catechin, epicatechin, epicatechin-3-O-gallate, tannins, and anthocyanins [135]. Various studies have demonstrated the beneficial effects of OMWW polyphenols in the diet, as they help regulate oxidative stress [62]. Previous research also shows that diets supplemented with OMWW and GP improve the redox status of sheep [136].

The function of most phenolic compounds is to donate hydrogen atoms or electrons or to interrupt free radical chain reactions, thereby preventing metal ion chelation and down-regulating pro-oxidant enzymes [137]. The lower levels of ROMs observed in treated goats indicate that BPS can reduce free radical production and oxidative stress, and these reductions are associated with higher TAS levels. These findings are consistent with previous studies in goats supplemented with flavonoids [138,139] and in laying hens receiving mixtures of powdered plant leaves [76].

Active interactions between endogenous and exogenous antioxidants in scavenging free radicals have also been demonstrated [140]. The increase in blood vitamin E observed in our study may be due to the ability of BPS to enhance and preserve the endogenous antioxidant system by modulating oxidative metabolism and reducing free radical species. It has been suggested that polyphenols may: regulate cytochrome P450 and that α-hydroxylase in hepatocytes inhibits the activity of α-tocopherol [141]; protect tocopherols from the redox cycle and promote the regeneration of tocopheryl radicals [142,143]; and alter tocopherol-binding ability and/or the gene expression of the tocopherol transfer protein [144].

Milk oxidative status and stability depend on the balance between antioxidants and pro-oxidants, as well as on the presence of substances susceptible to oxidation, mainly PUFAs, which are particularly prone to degradation [130]. Therefore, an adequate amount of antioxidants is required to slow down oxidative processes. In goats, the transfer of dietary polyphenols into milk has been demonstrated [145,146], and this can enhance the nutraceutical properties of milk through the potential antioxidant, anti-inflammatory [147], anticancer [148], and antimicrobial [149] activities of polyphenols. In this study, the polyphenol content of milk was significantly higher in BPS-treated goats. Several studies report that the antioxidant activity of milk is directly related to its concentration of polyphenolic compounds [150,151,152]. Moreover, during polyphenol metabolism, glycosylation has been shown to protect them from ruminal degradation and improve their bioavailability [153].

The increased milk vitamin E levels in BPS-treated goats may result from the antioxidant activity of BPS at the blood level, which supports the endogenous antioxidant system, reduces oxidative stress markers, and consequently increases the availability of vitamin E for the mammary gland and for milk antioxidant activity. According to Martin et al. [154], vitamin E concentrations in blood and goat milk are closely linked. Vitamin E can improve the nutritional value and oxidative stability of milk by preventing the development of oxidized flavors, especially when PUFA levels are high [155].

Several studies have demonstrated the positive effects of polyphenols on milk oxidative stability [100,152], in agreement with the results of the present research.

The objective of the TBARS analysis was to assess potential lipid oxidation in milk and thus evaluate its quality and stability, given that TBARS are primary by-products of lipid peroxidation. Onjai-Uea et al. [156] demonstrated that polyphenols can decrease malondialdehyde concentrations in the blood and milk of lactating dairy goats. Polyphenols are capable of preventing lipid peroxidation reactions and TBARS formation by scavenging free radicals and halting peroxidation pathways, thus preventing chemical–physical changes and undesirable odors, flavors, and colors. In this study, TBARS levels were lower in the BPS group compared with the CON group. Furthermore, TBARS levels in goat milk paralleled those of blood markers, in agreement with Yang et al. [157]. This strong antioxidant activity can be attributed to the polyphenolic compounds present in the OMWW and GP components of BPS. According to Asli et al. [158], in the olive-pressing process (three-phase system), generally only about 2% of total phenols are transferred to olive oil, whereas 98% remain in OMWW. The antioxidant activity of OMWW is attributed to oleuropein, hydroxytyrosol, tyrosol, caffeic acid, p-coumaric acid, and verbascoside [27], which exhibit comparable or even superior antioxidant capacity to natural antioxidants such as vitamin C or Trolox [159]. Supplementing pig diets with OMWW has been shown to reduce TBARS levels and increase meat stability [37,114]. The antioxidant activity of GP, attributed to gallic, protocatechuic, vanillic, syringic, and caffeic acids, as well as catechin, epicatechin, resveratrol, quercetin, and kaempferol [160], also contributes to reducing TBARS levels [161,162], thereby extending milk oxidative stability and improving its quality. Overall, our results provide evidence that BPS may transfer polyphenols into milk and enhance the antioxidant activity of lactating dairy goats.

## 5. Conclusions

This study is one of the few where a silage was formulated using raw waste by-products from the cereal, olive oil, wine, and dairy chains, and validated its use as a dietary supplement for lactating dairy goats. Based on the results, it can be concluded that a combination of raw agro-industrial by-products—WS (40%), GP (20%), OMWW (5%), and CW (35%)—produced a suitable silage in terms of nutritional composition and antioxidant potential. The BPS production in cylindrical bale silos (50 kg) is a practical and promising technique, as it preserves nutritional composition over time. The BPS was tested at a 30% inclusion level (dry-matter basis) in the total ration of lactating goats, resulting in increased milk yield. Dietary BPS supplementation had a positive influence on the oxidative status (TAS, ROMs, and vitamin E levels) and metabolic parameters related to lipid and immune profiles of goats. Furthermore, BPS supplementation improved goat milk quality, including the fatty acid profile, vitamin E content, reduced cholesterol levels, and improved oxidative stability. These findings indicate that milk quality can be improved through the transfer of polyphenolic compounds from BPS and their antioxidant activity, resulting in polyphenol and antioxidant enrichment, nutritional improvement, and greater oxidative stability of milk. The silage (BPS) can be a promising dietary strategy to improve goat milk quality and support the sustainability of milk production. Further research is needed to clarify the mechanisms of action of the antioxidant compounds derived from GP and OMWW in BPS and to determine the interactions between the components of each raw material and their effects on animal welfare, productive performance, and dairy product quality.

## Figures and Tables

**Table 1:** Ingredients, nutrient composition, and daily amounts of feed and nutrients offered in the experimental (BPS) and control diets fed to lactating goats (DM basis).

Ingredients (g/kg Diet DM)	CON	BPS
Oat hay	804.02	491.90
Corn flour	130.65	135.70
Soy bean meal 44% CP	55.28	63.30
Silage	/	300.00
Vitamin and mineral premix *	10.05	9.10
**Chemical composition of the experimental diets (g/kg)**		
Crude protein	101.2	102.2
Ether extract	49.6	58.3
Organic matter	932.1	939.9
Starch	202.9	258.6
Crude fiber	260.0	280.0
NDF	547.0	539.6
ADF	290.0	315.5
ADL	97.0	102.0
NFE	521.3	499.4
Metabolizable energy (MJ/kg DM)	7.5	8.8
DM kg/day	0.995	1.008

CON: control group receiving basal diet without experimental silage; BPS: experimental group receiving agro-industrial by-product silage (wheat straw 40% + grape pomace 20% + cheese whey 35% + olive mill wastewater 5%) supplemented at 30% (based DM) of the total basal ration. NDF: neutral detergent fiber; ADF: acid detergent fiber; ADL: acid detergent lignin; NFE: nitrogen-free extract (starch and sugar). Metabolizable energy estimate in accordance with INRA [51]: ME (MJ/kg DM) = 0.031 × CP + 0.148 × EE + 0.122 × NFE + 0.045 × CF, where: DM = dry matter; CP = crude protein; EE = ether extract; NFE: nitrogen free extract; CF = crude fiber. * Premix: nutritional supplement with vitamin (A, D3, E), mineral and plant extract in alcohol solution. Base ingredients: soy (400 g/kg), rapeseed cake (200 g/kg), maize flour (120 g/kg), wheat sharp (40 g/kg), sugarcane molasses (25 g/kg), D-L-methionine (10 g/kg), calcium carbonate (8 g/kg), di-calcium phosphate anhydrous (6 g/kg), and NaCl (5 g/kg).

**Table 2:** Chemical composition (% of dry matter) and pH at D0 and D40 (maturation time) and fermentative quality at D40 of the agro-industrial by-product silage (BPS) included in the experimental diet.

Chemical Composition	D0	D40	SEM	*p* Value
Dry matter	51.22	50.95	0.073	0.07
Crude protein	5.53	5.45	0.026	0.10
Ether extract	2.04	1.99	0.014	0.08
Ash	6.47	6.21	0.13	0.18
NDF	60.66	62.64	1.00	0.19
ADF	43.42	44.24	0.45	0.21
ADL	8.23	8.53	0.16	0.20
TPC, mg GAE g^−1^ DM	1.62	2.57	0.021	<0.01
**Fermentative characteristics** (x ± SE)				
pH	5.59 ± 0.11	3.99 ± 0.11		<0.01
Lactic acid, g kg^−1^ DM	-	26.50 ± 2.30		
Acetic acid, g/kg^−1^ DM	-	14.51 ± 1.46		
Propionic acid, g/kg^−1^ DM	-	0.03 ± 0.01		
Isobutyric acid, g/kg^−1^ DM	-	0.01 ± 0.01		
Butyric acid, g/kg^−1^ DM	-	1.98 ± 0.28		
Total VFA, g/kg^−1^ DM	-	18.35 ± 2.09		
Total acids, g/kg^−1^ DM	-	44.86 ± 1.66		
% total VFA/total acids	-	41.19 ± 4.50		
Fleig’s score		146.64		

BPS: agro-industrial by-product silage including: wheat straw 40%, grape pomace 20%, cheese whey 35%, and olive mill wastewater 5% (on a fresh-matter basis). - = not detected; NDF: neutral detergent fiber; ADF: acid detergent fiber; ADL: acid detergent lignin. TPC: total phenol content GAE, mg gallic acid equivalents per g of dry matter (DM). VFA: sum of acetic, propionic, isobutyric and butyric acids. Total acids: VFA plus lactic acid. Fleig’s score in accordance with Dong et al. [49].

**Table 3:** Milk yield, chemical composition, somatic cell count content (SCC), and fecal short chain fatty acids (SCFAs) of goat fed the experimental diets.

Chemical Composition	CON	BPS	SEM	*p* Value
Animals, *n*.	15	15		
Milk yield, g/d	782.23	969.81	53.79	0.048
Protein, %	2.89	3.07	0.092	0.231
Fat, %	3.27	3.36	0.117	0.318
Lactose, %	4.76	4.83	0.041	0.327
Cholesterol, mg/100 g	11.22	10.07	0.166	0.001
SCC, N × 10^3^/mL	386.01	271.430	59.27	0.045
**Fecal SCFA**				
Acetic acid, g kg^−1^ DM	7.23	7.42	0.212	0.575
Propionic acid, g kg^−1^ DM	2.79	4.05	0.129	0.001
Butyric acid, g kg^−1^ DM	2.33	3.40	0.130	0.001

CON: control group receiving basal diet without experimental silage; BPS: experimental group receiving agro-industrial by-product silage (wheat straw 40% + grape pomace 20% + cheese whey 35% + olive mill wastewater 5%, on a fresh-matter basis) supplemented at 30% (based DM) of the total basal ration. SCC: somatic cell count.

**Table 4:** Fatty acid profile in goat milk.

Fatty Acids (g/100 g FAME)	CON	BPS	SEM	*p* Value
C4:0; butyric	2.65	2.53	0.040	0.368
C6:0; caproic	2.57	2.54	0.030	0.157
C8:0; caprylic	2.70	2.09	0.022	0.189
C10:0; capric	8.03	7.37	0.058	0.065
C12:0; lauric	4.84	4.62	0.062	0.797
C14:0; myristic	9.83	9.81	0.188	0.778
C14:1; myristoleic	0.31	0.52	0.025	0.014
C15:0; pentadenoic	1.24	1.20	0.029	0.140
C16:0; palmitic	24.45	23.89	0.196	0.010
C16:1; palmitoleic	0.78	0.90	0.029	0.158
C18:0; stearic	13.07	13.54	0.078	0.025
C18:1 t11; trans-vaccenic	2.74	2.73	0.052	0.939
C18:1 cis-9; oleic	19.93	20.05	0.082	0.459
C18:2 n-6; linoleic	2.23	2.28	0.038	0.193
C18:2 n-3 cis-9, trans-11 CLA; rumenic	1.34	1.58	0.039	0.048
C18:3 n-3; linolenic	1.60	1.70	0.044	0.035
C20:0; arachidic	0.39	0.41	0.025	0.715
C20:4 n-6; arachidonic	0.38	0.44	0.020	0.054
C20:5 n3; eicosapentanoic	0.41	0.55	0.024	0.022
C22:6 n3; docosahesanoic	0.44	0.53	0.022	0.037
SFA, saturated	69.77	67.72	0.167	0.046
MUFA, monounsaturated	23.76	25.20	0.106	0.132
PUFA, polyunsaturated	6.47	7.08	0.114	0.008

CON: control group receiving basal diet without experimental silage; BPS: experimental group receiving agro-industrial by-product silage (wheat straw 40% + grape pomace 20% + cheese whey 35% + olive mill wastewater 5%, on a fresh-matter basis) supplemented at 30% (based on DM) of the total basal ration.

**Table 5:** Blood proteic, lipidic, hepatic, and immunological profiles in goats.

	CON	BPS	SEM	*p* Value
Animals, *n*.	15	15		
Total protein, g/dL	5.39	5.51	0.728	0.341
Triglycerides, mg/dL	21.62	21.25	0.847	0.618
Total cholesterol, mg/dL	109.52	103.69	2.923	0.032
HDL cholesterol, mg/dL	103.63	110.17	3.480	0.048
Alkaline phosphatase (ALP), IU/L	107.32	108.49	15.04	0.293
Aspartate aminotransferase, (AST) IU/L	75.94	79.02	5.022	0.211
Alanine aminotransferase (ALT) IU/L	16.07	15.91	1.145	0.184
TNF-α, pg/mL	13.57	10.24	0.850	0.041
IL-6, pg/mL	14.96	13.35	0.971	0.152
IL-1β, pg/mL	35.13	16.88	0.962	0.009

CON: control group receiving basal diet without experimental silage; BPS: experimental group receiving agro-industrial by-product silage (wheat straw 40% + grape pomace 20% + cheese whey 35% + olive mill wastewater 5%, on a fresh-matter basis) supplemented at 30% (based on DM) of the total basal ration. HDL: high-density lipoprotein; TNF-α = tumor necrosis factor; IL-1β = interleukine-1β; IL-6 = interleukine-6.

**Table 6:** Effect of PBS on antioxidant activity in blood and milk of goats.

	CON	BPS	SEM	*p* Value
Animals, *n*.	15	15		
Blood oxidative status				
TAS, μmol Trolox Eq/L	370.50	450.63	23.31	0.048
ROMs, UCarr	193.711	138.292	6.985	0.006
Vitamin A, mg/L	0.13	0.11	0.006	0.257
Vitamin E, mg/L	1.301	1.432	0.091	0.027
Milk oxidative status				
Total phenol content, mg GAE/L	53.18	87.44	0.129	0.027
Vitamin A, mg/L	0.14	0.21	0.001	0.381
Vitamin E, mg/L	0.097	0.23	0.021	0.032
TBARS, μmol/L	0.825	0.688	0.039	0.023

CON: control group receiving basal diet without experimental silage; BPS: experimental group receiving agro-industrial by-product silage (wheat straw 40% + grape pomace 20% + cheese whey 35% + olive mill wastewater 5%, on a fresh-matter basis) supplemented at 30% (based on DM) of the total basal ration. ROMs = reactive oxygen metabolites; TBARS: thiobarbituric acid reactive substances expressed as micromoles of MDA (malondialdehyde) per L of milk.

## Data Availability

Data are contained within the article.
